# Mitigating Interfacial Degradation by Tuning the Diluent–Anion Affinity for Long-Cycling Lithium Metal Batteries

**DOI:** 10.3390/ma19122605

**Published:** 2026-06-17

**Authors:** Hongcheng Wu, Jiangnan Ran, Youxian Dou, Dalin Yang, Guangye Wu, Qiang Zheng

**Affiliations:** 1 School of Materials Science and Engineering, Shanghai University, Shanghai 200444, China; hongchengwu@shu.edu.cn (H.W.); 2472996517@shu.edu.cn (J.R.); 2 I-Lab, Suzhou Institute of Nano-Tech and Nano-Bionics, Chinese Academy of Sciences, Suzhou 215123, China; yxdou2025@sinano.ac.cn (Y.D.); dlyang2025@sinano.ac.cn (D.Y.)

**Keywords:** lithium metal batteries, ionic liquids, high-safety electrolytes, localized high-concentration electrolytes

## Abstract

**Highlights:**

- TeCA with weak intermolecular interaction achieves a compressed solvation structure by mitigating diluent–FSI^−^ interaction.
- TeCA-LHCE shows good flame retardancy and cost advantages compared with commercial carbonate electrolytes.
- TeCA-LHCE electrolyte can reduce the fluorine content in the electrolyte, thereby better complying with PFAS regulations.
- TeCA-LHCE enables 99.23% average CE over 500 cycles in Li||Cu cell and stable cycling over 800 h in Li||Li symmetric cells.
- Full cells employing TeCA-LHCE achieve improved cycling stability at 4.3 V and wide-temperature adaptability.

**Abstract:**

Ionic liquid-based localized high-concentration electrolytes, leveraging their intrinsically nonflammable safety characteristics and wide electrochemical windows, have emerged as strong contenders for next-generation lithium metal battery electrolytes. However, because such systems are anion-rich, the electrolyte bulk phase tends to form solvation structures dominated by bulky anionic clusters along with an excess of free anions, which triggers persistent and uncontrollable anion decomposition at the interphase. To address this issue, we adopt a strategy of constructing a compressed solvation structure by introducing a weakly interacting chlorinated diluent (TeCA), which helps form a compact solvation environment and alleviates excessive anion decomposition at electrode interphases. In this work, 1,1,2,2-tetrachloroethyl acetate (TeCA) was introduced as a weakly coordinating chlorinated diluent into an ionic-liquid localized high-concentration electrolyte (LHCE) to regulate the Li^+^-FSI^−^ solvation environment. By combining Raman spectroscopy, molecular dynamics simulations, and electrochemical characterization, the TeCA-LHCE system was found to exhibit altered ion-cluster configurations, improved oxidation tolerance, and enhanced interfacial stability under high-voltage conditions. The as-prepared TeCA-LHCE electrolyte presents improved electrochemical performance in comparison with TTE-LHCE and the baseline electrolyte (BE). The Li||Cu half-cell employing TeCA-LHCE achieved a high Coulombic efficiency above 99% over 500 cycles and formed a uniform and dense lithium deposition layer without obvious dendritic growth. When paired with a high-loading NCM811 cathode (10 mg cm^−2^), the TeCA-LHCE-based Li||NCM811 full cell delivered significantly improved cycling stability and rate capability under a high cutoff voltage of 4.3 V.

## 1. Introduction

With the rapid development of electric vehicles and portable electronic devices [1,2,3], the demand for high-energy-density batteries has continuously increased. Lithium metal has attracted extensive attention due to its ultrahigh theoretical specific capacity and low electrode potential. When paired with high-voltage cathode materials, the resulting battery system is expected to achieve an energy density exceeding 500 Wh kg^−1^ [4,5]. However, the intrinsic instability of the electrode/electrolyte interphase [6,7] and the uncontrollable growth of lithium dendrites severely hinder the practical development of lithium metal batteries [8,9]. As a key component of lithium metal batteries, the electrolyte plays a crucial role in determining battery performance. Conventional carbonate-based electrolytes possess a narrow electrochemical stability window and are inherently highly flammable, posing significant challenges for the large-scale commercialization of lithium metal batteries [10]. In recent years, ionic liquids, as room-temperature molten salts, have attracted widespread interest owing to their wide electrochemical stability window [11], good SEI-forming capability [12], and intrinsically nonflammable physicochemical properties. Although high-concentration electrolytes formed by combining ionic liquids with highly concentrated lithium salts exhibit robust interfacial film-forming capability, their high viscosity results in low ionic conductivity and poor wettability toward the micropores within porous electrodes. Therefore, introducing low-viscosity diluents has become a mainstream strategy in current research [13].

In recently developed localized high-concentration electrolytes (LHCEs) [14,15], diluents such as bis(2,2,2-trifluoroethyl) ether (BTFE) [16,17], 1,1,2,2-tetrafluoroethyl-2,2,3,3-tetrafluoropropyl ether (TTE) [18,19], and 3,3,4,4,5,5-hexafluorotetrahydropyran (HFTHP) [20] are commonly used. These diluents effectively facilitate the formation of a favorable interphase film and suppress lithium dendrite growth. However, most of these diluents contain fluorinated groups with strong electron-withdrawing effects, which generate weak interactions with bis(fluorosulfonyl)imide anion (FSI^−^), thereby perturbing the original anion-rich solvation structure. In addition, these diluents are highly fluorinated ether-based alkanes and are generally associated with high costs. Moreover, a series of environmental regulations and restrictions targeting per- and polyfluoroalkyl substances (PFAS) [21,22,23] have recently been introduced in Europe and the United States, further limiting their practical applications. Therefore, the development of novel environmentally compatible diluents is urgently needed.

In this work, a compressed solvation structure refers to a solvation environment with a reduced first-shell radius and a higher fraction of coordinated FSI^−^ anions, resulting in fewer free anions and smaller ion-cluster dimensions. This study focuses on chlorinated diluents and identifies a short-chain, highly chlorinated novel diluent: 1,1,2,2-tetrachloroethyl acetate (TeCA). Substituting fluorine atoms with chlorine atoms can reduce the overall fluorine content of the electrolyte, showing good potential to adapt to current PFAS regulatory trends. Furthermore, the introduction of chlorinated diluents effectively enhances the high-voltage stability of the electrolyte system. Previous studies have demonstrated the feasibility of chlorinated weakly coordinating diluents in LHCE systems. In the present work, we further explore the tetrachlorinated TeCA molecule within an ionic-liquid-based LHCE environment. Compared with previously reported dichlorinated diluents, the increased degree of chlorination of TeCA may affect ion-ion interactions in a distinct manner. First, the strong electronegativity and electron-withdrawing effect of chlorine atoms lower the electron cloud density of the molecule itself [24]; multiple chlorine atoms further reduce its electrostatic potential, thereby diminishing electrostatic interactions with other molecules and consequently modulating the solvation structure in the electrolyte [25]. Second, chlorinated diluents generally exhibit low volatility and high flash points, inhibiting the generation of flammable vapors under thermal runaway conditions and thus significantly reducing the flammability of the electrolyte [26,27].

This work systematically investigates the influence of diluent chemical structure on the solvation chemistry and electrochemical performance of ionic liquid-based localized high-concentration electrolytes (IL-LHCEs) for high-voltage lithium metal batteries. Density functional theory (DFT) calculations reveal that TeCA possesses a lower maximum electrostatic potential (ESPmax) than TTE, indicating weaker electrostatic interactions with FSI^−^ anions and thus less disruption to the Li^+^ solvation sheath. Scanning electron microscopy (SEM) observations indicate that lithium deposited in TeCA-LHCE exhibits a dense and uniform morphology without obvious dendritic structures, whereas loose and porous lithium deposits are observed in the comparison electrolytes. The Li||NCM811 full cell assembled with this electrolyte and a high-areal-loading NCM811 cathode (10 mg cm^−2^) maintained stable cycling over 250 cycles with a capacity retention of 66%, demonstrating promising practical application potential.

## 2. Materials and Methods

### 2.1. Materials Preparation and Battery Assembly

To ensure the reliability and reproducibility of the experimental data, all materials were rigorously pretreated, and battery assembly as well as sample preparation were carried out in a high-purity argon-filled glovebox. All electrolyte formulations were prepared independently in triplicate. Specifically, 3 Å molecular sieves were activated in a muffle furnace at 300 °C for 72 h. N-Methyl-N-propylpyrrolidinium bis(fluorosulfonyl)imide (Pyr_13_FSI, 99.9%, Changde Dadu New Materials Co., Ltd., Changde, China) and lithium bis(fluorosulfonyl)imide (LiFSI, 99.9%, Suzhou Gudina New Energy Technology Co., Ltd., Suzhou, China) were vacuum-dried at 100 °C for 24 h prior to use. TeCA (99%, Adamas-Beta Reagents Co., Ltd., Shanghai, China) and TTE (98%, Adamas-Beta Reagents Co., Ltd., Shanghai, China) were dried over activated molecular sieves at room temperature for 48 h before use. Lithium foil with a thickness of 450 μm was employed as the anode, and NCM811 with an areal loading of 10 mg cm^−2^ served as the cathode material. CR2025 coin cells were assembled in an argon-atmosphere glovebox with water and oxygen contents both below 2 ppm, with 80 μL of electrolyte injected into each cell. All electrochemical measurements were conducted at 25 °C. TeCA-LHCE and TTE-LHCE were prepared by mixing LiFSI, Pyr_13_FSI, and the corresponding diluent at a molar ratio of 1:2:3 in sequence, followed by magnetic stirring for 30 min at room temperature. The molar ratio of LiFSI:Pyr_13_FSI:diluent = 1:2:3 was adopted for both TeCA-LHCE and TTE-LHCE. The baseline carbonate electrolyte (BE) used in this work was commercial LiPF_6_-based carbonate electrolyte (1 M LiPF_6_ in EC/DMC = 1:1 vol%), which was used as received without further treatment.

### 2.2. Characterization Methods

The morphology of lithium deposition surfaces was characterized using a cold-cathode field-emission scanning electron microscope (SEM, Hitachi S4800, Hitachi High-Technologies Corp., Tokyo, Japan). The chemical compositions of the cathode-electrolyte interphase were analyzed by X-ray photoelectron spectroscopy (XPS, Thermo Scientific K-Alpha^+^, Thermo Fisher Scientific, Waltham, MA, USA) using an Al Kα X-ray source (12 kV, 72 W). Lithium anode samples after cycling were rinsed with dimethyl carbonate (DMC), dried in the argon-filled glovebox, and then transferred to characterization equipment via a vacuum transfer vessel to avoid exposure to air and moisture.

### 2.3. Electrochemical Measurements

The ionic conductivity of the electrolytes was measured using a Mettler Toledo S400 conductivity meter (Mettler-Toledo, Greifensee, Switzerland) equipped with an InLab 741-ISM electrode (Mettler-Toledo, Greifensee, Switzerland). Electrochemical impedance spectroscopy (EIS) was performed on a BioLogic VMP-300 electrochemical workstation (BioLogic, Fontaine, France). Distribution of relaxation time (DRT) analysis of the EIS data was conducted using the Bayesian fitting method available on the DRTtools.com website, with a tested frequency range of 10^−2^–10^6^ Hz, a fixed regularization coefficient of 0.01, and standard fitting boundary conditions. All key electrochemical results (including linear sweep voltammetry, electrochemical impedance spectroscopy, and battery cycling performance) were obtained from at least three independently assembled cells, each using an independently prepared electrolyte batch. All characterization and electrochemical tests were repeated using three independent batches of electrolytes and cells. The curves shown in the manuscript are representative results from these repeated tests, and the data exhibited good reproducibility.

### 2.4. Computational Methods

Molecular dynamics (MD) simulations were performed using the Forcite module of Materials Studio 2020. Electrolyte models based on the molar ratios of LiFSI/Pyr_13_FSI/diluent were constructed, and the COMPASS III force field was employed to describe molecular interactions. Each system was first equilibrated in the NPT ensemble (298.15 K, 1.0 × 10^−4^ GPa) for 0.5 ns using Nosé–Hoover thermostat control with a van der Waals cutoff radius of 12.5 Å. Subsequently, a 1 ns production run was carried out in the NVT ensemble (298.15 K) with a time step of 1 fs, and the NVT trajectory was used to analyze the solvation structures. Quantum chemical calculations were conducted using Gaussian 16 at the B3LYP-D3/def2-SVP level, and RESP atomic charges were computed with the Multiwfn 3.8 program.

## 3. Results and Discussion

Compared with widely reported fluorinated alkane diluents, chlorinated alkane diluents feature lower costs [13] and simpler molecular structures. TeCA, a small-molecule and highly halogenated compound, was selected as the diluent in this work. This compound shows relatively stable physicochemical properties and weak reactivity toward lithium metal under test conditions [24]. Currently, TTE is widely used as a diluent in localized high-concentration electrolytes owing to its good wettability, decent electrical conductivity, and relatively wide electrochemical window [28,29,30]. To clarify the inherent discrepancy in bulk ion-transport behaviors between two electrolytes, we quantified their key physicochemical properties and lithium-ion transference numbers at 25 °C. Relevant experimental data including ionic conductivity, viscosity, density, electrode contact angle, and Bruce–Vincent-derived t_Li+_ values are summarized in Figure S1. Therefore, in this study, TTE was selected as a reference system to compare the properties of chlorinated and fluorinated diluents.

**Figure 1:**
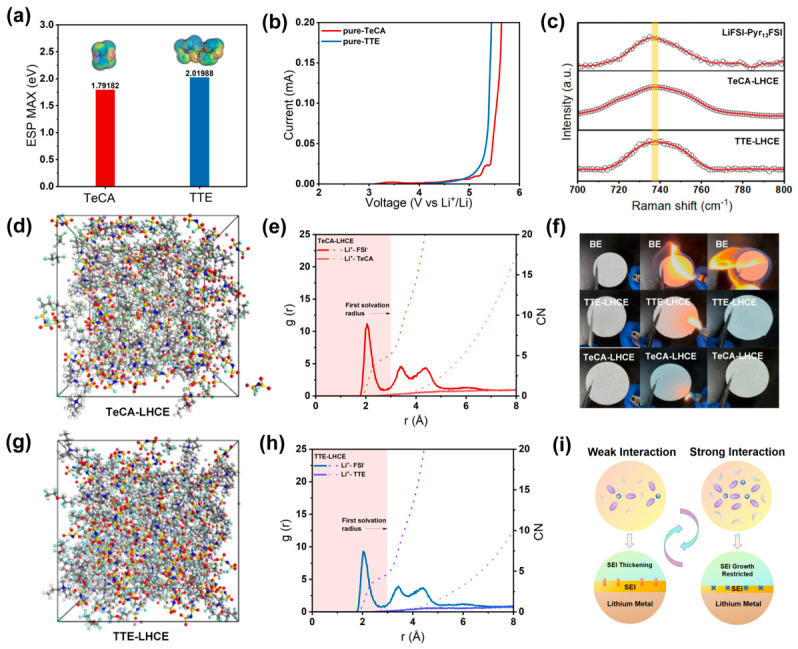
(**a**) Maximum electrostatic potential (ESP) of TeCA and TTE molecules calculated based on density functional theory (DFT). (**b**) Linear sweep voltammetry (LSV) measurements of pure TeCA and TTE molecules to compare their anodic oxidation resistance. The potential was continuously scanned from 2.0 V up to 6.0 V (vs. Li^+^/Li) at a fixed sweep rate. (**c**) Raman spectra of the pristine LiFSI-Pyr_13_FSI ionic liquid electrolyte, TeCA-LHCE, and TTE-LHCE in the range of 700~800 cm^−1^. (**d**,**g**) Molecular dynamics simulation snapshots of the TeCA-LHCE and TTE-LHCE electrolyte systems. (**e**,**h**) Radial distribution functions and corresponding coordination numbers between Li^+^, FSI^−^ anions, and diluent molecules. (**f**) Comparative flammability tests of the baseline electrolyte (BE), TTE-LHCE, and TeCA-LHCE. (**i**) Schematic illustration of the influence of diluent-anion interaction strength on Li^+^ solvation behavior and interfacial evolution.

To elucidate the intrinsic differences between TeCA and TTE in terms of molecular polarity and electrochemical stability, density functional theory (DFT) calculations were performed, including electrostatic potential (ESP) analysis and frontier molecular orbital calculations. As shown in Figure 1a, TeCA exhibits a significantly lower maximum electrostatic potential (ESPmax = 1.79182 eV) than TTE (2.01988 eV), indicating weaker electrostatic interactions with anions and thus reduced disruption to the Li^+^ solvation sheath. To evaluate the intrinsic electrochemical stability of the two diluent molecules, linear sweep voltammetry (LSV) was performed on pure TeCA and TTE (Figure 1b). The results show that pure TeCA exhibits a higher oxidation onset potential, which suggests that TeCA may deliver better oxidation resistance than TTE. Accordingly, it is speculated that the LHCE formulated with TeCA may have a wider electrochemical stability window. Subsequently, two electrolytes, TeCA-LHCE and TTE-LHCE, were prepared with a molar ratio of LiFSI:Pyr_13_FSI:diluent = 1:2:3. Raman spectroscopy (Figure 1c) was used to characterize the pristine ionic liquid electrolyte (LiFSI-Pyr_13_FSI), TeCA-LHCE, and TTE-LHCE, focusing on the characteristic S-N-S stretching vibration of the FSI^−^ anion in the 700~800 cm^−1^ region [31,32]. To analyze the FSI^−^ solvation configuration variation upon different diluent addition, Raman spectra of LiFSI-Pyr_13_FSI, TeCA-LHCE and TTE-LHCE were peak-fitted into three components of SSIP, CIP and AGG, and the corresponding peak splitting results and component percentage statistics are provided in Figure S2. All three electrolytes exhibit similar peaks at approximately 740 cm^−1^. Since the solvent in the electrolyte system is an ionic liquid, which is mainly composed of cations and anions, and neither the cations nor the diluent participates in the coordination of lithium ions, the Raman results imply that the primary solvation sheaths of the three electrolytes are all dominated by FSI^−^ anions.

We further investigated the solvation structures of the two systems using molecular dynamics (MD) simulations (Figure 1d,g) [33,34]. To ensure the robustness of the experimental findings, three additional replicate experiments were conducted for each system, and the results proved to be reliable (Figure 1, Figures S3 and S4). In the TeCA-LHCE system (Figure 1e), the radial distribution function (RDF) of Li^+^-FSI^−^ exhibits a pronounced sharp peak at ~2.0 Å, corresponding to the first solvation shell of Li^+^; the coordination number curve rises rapidly within the solvation radius, yielding a coordination number (CN) of 4.53, which, combined with published literature [35], implies that a large number of FSI^−^ anions enter the primary solvation sheath. Meanwhile, the Li^+^-TeCA RDF shows almost no signal within the first solvation shell, demonstrating that TeCA scarcely participates in the primary solvation of Li^+^. In contrast, in the TTE-LHCE system (Figure 1h), the main peak of Li^+^-FSI^−^ also appears at ~2.0 Å but with lower intensity than that in TeCA-LHCE; the FSI^−^ coordination number increases more slowly, with a Li^+^-FSI^−^ coordination number of 4.22, which is lower than the value of 4.53 in the TeCA system, indicating that some anions do not enter the first solvation shell of Li^+^ and instead exist outside the solvation sheath as free anions or unstable anionic clusters [36]. Furthermore, statistical analysis reveals that the average first-solvation-shell size in the TTE-LHCE system is slightly larger than that in TeCA-LHCE (2.89 Å vs. 2.73 Å). The low coordination number indicates that a portion of FSI^−^ anions remains unpaired in the electrolyte. These uncoordinated anions exhibit poor oxidative stability [37], leading to their decomposition at the positive electrode and consequently increasing the rate of electrolyte depletion [38].

To evaluate flame-retardant safety, direct ignition tests were conducted to compare the combustion behaviors of a commercial carbonate-based electrolyte (BE), TTE-LHCE, and TeCA-LHCE (Figure 1f). The BE ignited rapidly upon exposure to an open flame and burned violently, indicating poor flame retardancy. Both TTE-LHCE and TeCA-LHCE showed no continuous combustion after exposure to an open flame, presenting obvious flame-retardant behavior. Finally, Figure 1i schematically illustrates the interfacial evolution process of different diluents on the surface of the lithium metal anode. Experimental phenomena show that strongly interacting diluents (e.g., TTE) tend to form anion-rich solvation sheaths. We infer that these anions may undergo uncontrolled decomposition on the anode surface and further lead to interfacial degradation. In contrast, weakly interacting diluents (e.g., TeCA) retain a large number of anions in the solvation sheath, reducing the number of free anions and the size of the solvation clusters. This makes the interfacial reaction more controllable, leading to the formation of a dense, uniform, and robust SEI layer, which likely effectively suppresses side reactions.

**Figure 2:**
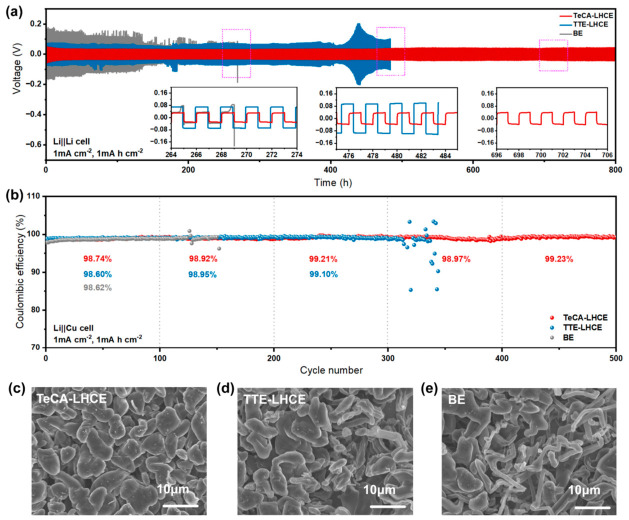
(**a**) Long-term cycling voltage-time profiles of Li||Li symmetric cells at a current density of 1 mA cm^−2^ and an areal capacity of 1 mAh cm^−2^. (**b**) Coulombic efficiency (CE) of Li||Cu asymmetric cells as a function of cycle number at a current density of 1 mA cm^−2^ and an areal capacity of 1 mAh cm^−2^. (**c**–**e**) Scanning electron microscopy (SEM) images of the surface morphologies of lithium deposited on copper current collectors in the three electrolyte systems.

In order to investigate the compatibility of the electrolytes with the anode side, Li||Li symmetric cells were assembled (Figure 2a). Under a current density of 1 mA cm^−2^ and an areal capacity of 1 mAh cm^−2^, the TeCA-LHCE system maintained a low and stable overpotential throughout 800 h of cycling without obvious polarization fluctuations, demonstrating improved lithium metal compatibility. In contrast, the BE system exhibited significant overpotential fluctuations and continuous overpotential growth starting from the early stage of cycling, indicating severe interfacial instability. Although the TTE-LHCE system showed relatively low polarization during the initial cycling stage, distinct voltage spikes and fluctuations appeared after approximately 400 h, suggesting a sharp increase in interfacial resistance. The enlarged voltage profiles shown in the inset clearly compare the voltage hysteresis at different stages, further corroborating that TeCA-LHCE can maintain stable lithium plating/stripping behavior.

Subsequently, to evaluate the lithium plating/stripping reversibility of the electrolytes, Li||Cu cells were assembled using different electrolytes and cycled at a current density of 1 mA cm^−2^ and an areal capacity of 1 mAh cm^−2^ (Figure 2b). The TeCA-LHCE system maintains a relatively stable and high Coulombic efficiency over 500 cycles, which indicates improved reversibility of lithium deposition and stripping under test conditions. In comparison, the Coulombic efficiency of the BE system remained below 99% with obvious fluctuations from the initial stage, whereas the TTE-LHCE system exhibited a pronounced decline and severe fluctuations after 300 cycles, indicating significantly deteriorated lithium deposition reversibility. The annotated average Coulombic efficiencies at representative cycling stages further quantify the performance differences among the three systems. Furthermore, 3 mAh cm^−2^ of lithium metal was deposited onto copper substrates to investigate the morphologies of lithium deposits formed in different electrolytes. The galvanostatic Li plating/stripping voltage curves of Li||Cu half-cells at the 100th and 200th cycles are summarized for TeCA-LHCE, TTE-LHCE and BE in Figure S5. As shown in Figure 2c–e, the deposited lithium in the TeCA-LHCE system exhibits a uniform and dense morphology with regular bulk-like structures, and no obvious dendrites or porous features are observed. In the TTE-LHCE system, partially irregular rod-like and needle-like protrusions are observed, reflecting uneven local current distribution. In contrast, the BE system forms disordered needle-like and flocculent lithium dendrites together with porous structures, which can readily induce interfacial side reactions and safety hazards. These morphological differences are consistent with the electrochemical results, suggesting that TeCA-LHCE may suppress lithium dendrite growth to a certain extent, thereby supporting the long-term cycling stability of lithium metal anodes.

**Figure 3:**
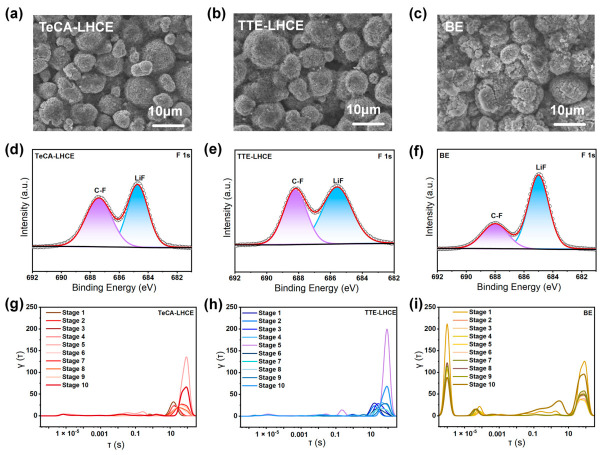
(**a**–**c**) Scanning electron microscopy (SEM) images of the surface morphologies of NCM811 cathodes after 100 cycles in the TeCA-LHCE, TTE-LHCE, and baseline electrolyte (BE) systems. (**d**–**f**) XPS F 1s spectra of the NCM811 cathode surfaces in the three electrolyte systems. (**g**–**i**) Distribution of relaxation time (DRT) fitting curves obtained from in situ electrochemical impedance spectroscopy (EIS) measurements of the three electrolyte systems after cycling in Li||NCM811 full cells.

To investigate the cathode-side performance of the designed electrolytes, Li||NCM811 cells were assembled using three different electrolytes and disassembled after 100 cycles to examine the cathode surface morphology. As shown in the SEM images in Figure 3a–c, after 100 cycles, NCM811 particles in the TeCA-LHCE system maintained intact spherical secondary structures with smooth surfaces and no obvious cracks, pulverization, or byproduct deposition, indicating that this electrolyte relatively alleviates structural degradation of the cathode under high-voltage conditions. In contrast, particles in the TTE-LHCE system exhibited slight thickening of the surface coating layer and increased roughness, whereas those in the BE system suffered severe structural degradation, with surfaces covered by substantial byproducts and blurred particle boundaries, reflecting the severe interfacial side reactions of conventional carbonate-based electrolytes. X-ray photoelectron spectroscopy (XPS) was subsequently conducted on the cycled cathode surfaces to analyze the composition of the interfacial layer (Figure 3d–f). All spectra can be deconvoluted into a LiF peak at 684.8 eV [39,40] and a C-F bond peak at ~688.0 eV [41,42]. In the TeCA-LHCE system, the F 1s spectrum is dominated by the LiF peak with a relatively weak C-F peak, indicating that the interfacial layer is primarily composed of comparatively stable inorganic LiF with limited organic byproducts. In the TTE-LHCE system, the relative intensity of the C-F peak slightly increases. Notably, although the TeCA-LHCE electrolyte has a significantly lower fluorine content than TTE-LHCE, the two systems exhibit comparable fluorine levels in the surface products. This phenomenon implies that TeCA-LHCE may facilitate the formation of inorganic-rich CEI derived from FSI^−^ anions.

In the BE system, the LiF peak intensity is also high, indicating the presence of abundant products from excessive FSI^−^ anion decomposition at the electrolyte interface. XPS Cl 2p spectra of cycled electrodes were measured to clarify the decomposition behavior of the chlorine-containing TeCA solvent, and the original and peak-deconvoluted spectra for the anode and cathode are displayed in Figure S6. In addition, the C 1s spectra (Figure S7) of the BE system show pronounced C-O signals on the surface of cycled cathodes, which arise from the severe decomposition of organic solvent components. This result demonstrates that the decomposition of free solvent molecules is dominant in BE-based cells, which is regarded as one of the key contributing factors to the relatively inferior cycling stability of the corresponding cells. In contrast, both TTE-LHCE and TeCA-LHCE show weaker C-O peaks and stronger S-O signals (Figure S7), indicating that the interfacial layer is mainly derived from FSI^−^ anion decomposition. As sulfur in this system comes exclusively from the FSI^−^ anion, a stronger characteristic signal corresponds to more extensive decomposition of FSI^−^, whose decomposition products take part in interfacial film formation. The resultant interfacial film is dominated by anion decomposition products.

Electrochemical impedance at different stages of a charge–discharge process in full cells assembled with different electrolytes (Figures S8–S10) was further measured and transformed into distribution of relaxation time (DRT) spectra to quantitatively evaluate the evolution of interfacial impedance during cycling, where peaks at different time scales correspond to interfacial contact, charge transfer, and solid-state ion diffusion processes, respectively [43]. In situ EIS combined with DRT analysis was implemented to trace interfacial impedance evolution upon continuous cycling for full cells with TeCA-LHCE, TTE-LHCE and BE electrolytes; the sequential DRT curves covering 10 cycling stages are summarized in Figure S11. In the TeCA-LHCE system, the DRT peak intensities remain consistently low throughout cycling, with no emergence of new peaks, indicating a low interfacial resistance (Figure 3g). The TTE-LHCE system (Figure 3h) exhibits a clear increase in impedance peaks at later cycles, reflecting a continuous rise in charge-transfer resistance. In contrast, the BE system (Figure 3i) exhibits high interfacial resistance even in the early cycles, with multiple intense new peaks emerging upon prolonged cycling. This higher interfacial impedance corresponds to a thicker CEI. According to previous literature [44], this phenomenon is likely caused by the decomposition of unstable ester solvents. Linear sweep voltammetry (LSV) tests on TeCA-LHCE and TTE-LHCE reveal that TeCA-LHCE exhibits a more delayed oxidation onset, indicating higher oxidative stability (Figure S12). On the one hand, this is attributed to the intrinsically better redox stability of TeCA. We speculate that its lower ESPmax may weaken interactions with FSI^−^ anions. On the other hand, the lower ESPmax of TeCA compared with TTE contributes to weaker interactions with FSI^−^ anions, enabling enhanced coordination between FSI^−^ and Li^+^, reducing free anion content, which could reduce irreversible decomposition of anion clusters at the interface and improve the high-voltage tolerance of the electrolyte.

In conclusion, the TeCA-LHCE electrolyte forms a stable and low-impedance interphase dominated by inorganic LiF on the NCM811 cathode surface, indicating that TeCA-LHCE mitigates electrolyte decomposition, structural degradation, and impedance growth under high-voltage conditions, thereby significantly enhancing the cycling stability of the cathode.

**Figure 4:**
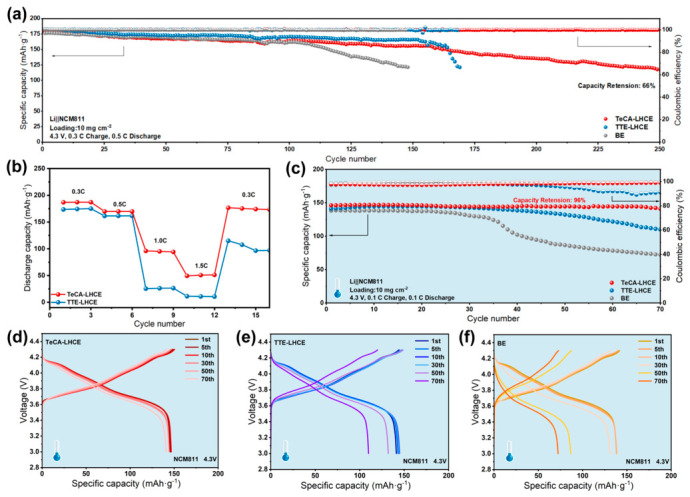
(**a**) Long-term cycling specific capacity and corresponding Coulombic efficiency of three electrolyte systems (TeCA-LHCE, TTE-LHCE, and BE) at a cutoff voltage of 4.3 V, a charge rate of 0.3 C, a discharge rate of 0.5 C, and a cathode areal loading of 10 mg cm^−2^. (**b**) Comparison of rate capability between the two electrolyte systems. (**c**) Long-term cycling performance of the three electrolyte systems under low-temperature conditions (indicated by the blue region in the figure) at a cutoff voltage of 4.3 V and a charge/discharge rate of 0.1 C. (**d**–**f**) Charge–discharge profiles of the TeCA-LHCE, TTE-LHCE, and BE systems, respectively, at different cycle numbers under low-temperature conditions.

Figure 4 systematically compares the electrochemical performance of Li||NCM811 full cells based on TeCA-LHCE, TTE-LHCE, and baseline electrolyte (BE) systems at room temperature (25 °C) and low temperature (−10 °C), comprehensively demonstrating the enhanced cycling stability, rate capability, and low-temperature adaptability enabled by the TeCA-LHCE electrolyte. In room-temperature long-term cycling tests (Figure 4a), under conditions of a 4.3 V cutoff voltage, 0.3 C charge/0.5 C discharge, and a cathode areal loading of 10 mg cm^−2^, the TeCA-LHCE system retained a high specific capacity after 250 cycles with a capacity retention of 66%, while maintaining a Coulombic efficiency close to 100%. In contrast, the TTE-LHCE system exhibited a pronounced capacity drop after approximately 150 cycles, whereas the BE system showed rapid capacity fading at the early stage, indicating that the TeCA-LHCE electrolyte suppresses the extent of interfacial side reactions under high voltage and maintains the structural stability of the cathode. Further analysis of the charge/discharge curves at different cycle numbers (Figure S13) reveals that the TeCA-LHCE system consistently maintains low polarization throughout cycling, whereas the TTE-LHCE system exhibits gradually increasing polarization in the later stages of cycling; the BE system also shows a significant rise in polarization. Rate capability tests of the two localized high-concentration electrolytes (Figure 4b) show that the TeCA-LHCE system exhibits high specific discharge capacities across a current density range of 0.3 C to 1.5 C, maintaining high capacity even at 1.5 C, and the capacity is almost fully recovered when the current is reduced back to 0.3 C, demonstrating improved rate performance. Rate-performance charge–discharge curves of Li||NCM811 full cells with TeCA-LHCE and TTE-LHCE from 0.3 C to 1.5 C are presented in Figure S14. Under low-temperature conditions (Figure 4c), long-term cycling results at a cutoff voltage of 4.3 V and a charge/discharge rate of 0.1 C indicate that the TeCA-LHCE system maintains high specific capacity and stable Coulombic efficiency over 70 cycles; the TTE-LHCE system exhibits significant capacity fading; and the BE system shows a sharp capacity decline. These results suggest that the TeCA-LHCE electrolyte maintains relatively stable interphase and ion transport properties even at low temperatures [45]. Low-temperature charge–discharge profiles at different cycle numbers (Figure 4d–f) provide further evidence for this conclusion: the TeCA-LHCE system shows minimal changes in curve shape and polarization during cycling, indicating the most stable electrochemical behavior. The TTE-LHCE system exhibits gradually increasing polarization, while the BE system shows a markedly shortened voltage plateau and significantly increased polarization, which shows the potential advantages of the TeCA-LHCE electrolyte under low-temperature and high-voltage conditions and suggests its promising practical potential.

## 4. Conclusions

Under the experimental conditions described in this work, we systematically compare the performance differences among TeCA-LHCE, TTE-LHCE, and baseline electrolyte (BE) in lithium metal batteries, revealing that TeCA-LHCE achieves synergistic interfacial stabilization of both the lithium metal anode and NCM811 cathode by regulating the electrolyte solvation structure. Theoretical calculations show that the TeCA molecule possesses a lower electrostatic potential, inhibiting the aggregation of bulky anionic clusters in the electrolyte, promoting the incorporation of anions into the Li^+^ solvation sheath, and forming a dense and stable CEI film dominated by low-impedance inorganic LiF on the NCM811 cathode surface. Electrochemical tests indicate that the TeCA-LHCE electrolyte enables over 500 cycles with a high Coulombic efficiency of up to 99.23% in Li||Cu cells, while inducing uniform and dense bulk-like lithium deposition that relatively mitigates dendrite growth. In high-voltage Li||NCM811 full cells, the TeCA-LHCE system retains a capacity of 66% after 250 cycles and also exhibits better low-temperature adaptability, maintaining stable capacity output even at −10 °C. In summary, the TeCA-LHCE electrolyte modulates the interfacial composition by regulating the ion coordination structure, restrains lithium dendrite growth at the anode, and maintains a comparatively stable interface at the high-voltage cathode, thereby providing an efficient and feasible electrolyte design strategy for practical lithium metal batteries with long cycle life and low operating temperature.

## Data Availability

The original contributions presented in this study are included in the article/Supplementary Material. Further inquiries can be directed to the corresponding authors.

## Supplementary Materials

The following supporting information can be downloaded at: https://www.mdpi.com/article/10.3390/ma19122605/s1. Figure S1. Physicochemical properties and Li^+^ transference numbers of TeCA-LHCE and TTE-LHCE electrolytes. (a) Ionic conductivity at 25 °C. (b) Viscosity at 25 °C. (c) Density at 25 °C. Representative contact angle images of (d) TeCA-LHCE and (e) TTE-LHCE on the electrode surface. (f) Quantified contact angle values. Chronoamperometry curves (main panels) and corresponding Nyquist plots (insets, before and after DC polarization) of Li symmetric cells with (g) TeCA-LHCE and (h) TTE-LHCE. The Li^+^ transference numbers (t_Li+_) calculated via the Bruce-Vincent method are labeled in each plot; Figure S2. Raman spectroscopy analysis of solvation structures in different electrolytes. Deconvoluted Raman spectra for (a) TeCA-LHCE, (b) TTE-LHCE, and (c) LiFSI-Pyr_13_FSI electrolytes. The spectra are fitted into three components corresponding to solvent-separated ion pairs (SSIP), contact ion pairs (CIP), and aggregates (AGG). (d) Relative percentages of SSIP, CIP, and AGG derived from peak deconvolution for the three electrolyte systems; Figure S3. Molecular dynamics (MD) simulation snapshots of equilibrated electrolyte systems. (a–c) Three independent parallel simulation runs with the TeCA-LHCE electrolyte. (d–f) Three independent parallel simulation runs with the TTE-LHCE electrolyte; Figure S4. Radial distribution functions (RDFs) and corresponding coordination numbers (CNs) from three independent molecular dynamics (MD) simulations. (a–c) RDF and CN for Li^+^-FSI^-^ and Li^+^-TeCA in the TeCA-LHCE electrolyte. (d-f) RDF and CN for Li^+^-FSI^-^ and Li^+^-TTE in the TTE-LHCE electrolyte. The pink shaded region denotes the first solvation radius of Li^+^; Figure S5. (a,b) Galvanostatic lithium plating/stripping voltage profiles of Li||Cu cells in the TeCA-LHCE, TTE-LHCE, and baseline electrolyte (BE) systems at the 100th and 200th cycles, respectively; Figure S6. XPS Cl 2p characterization of cycled anodes and cathodes in TeCA-LHCE and TTE-LHCE electrolytes. (a) Unfitted raw Cl 2p spectrum of a cycled anode from the TeCA-LHCE system. (b) Deconvoluted Cl 2p spectrum of a cycled cathode from the TeCA-LHCE electrolyte, showing resolved spin–orbit split 2p_1/2_ and 2p_3/2_ peaks. (c) Unfitted raw Cl 2p spectrum of a cycled anode from the TTE-LHCE system. (d) Deconvoluted Cl 2p spectrum of a cycled cathode from the TTE-LHCE electrolyte, with resolved 2p_1/2_ and 2p_3/2_ peaks; Figure S7. (a–c) Fitted O 1s XPS spectra of the NCM811 cathode surfaces in the BE, TTE-LHCE, and TeCA-LHCE systems, respectively. (d-f) Corresponding N 1s XPS spectra of the three electrolyte systems. (g–i) Fitted S 2p XPS spectra of the three electrolyte systems; Figure S8. (a–j) Nyquist plots of in situ electrochemical impedance spectra corresponding to different testing stages of the DRT fitting curves during conventional cycling (cutoff voltage of 4.3 V) of Li||NCM811 full cells with the TeCA-LHCE electrolyte system. The spectra consist of a semicircle in the high-frequency region, corresponding to interfacial contact and charge-transfer resistance, and an inclined line in the low-frequency region, corresponding to the solid-state ion diffusion process; Figure S9. (a–j) Nyquist plots of in situ electrochemical impedance spectra corresponding to different testing stages of the DRT fitting curves during conventional cycling (cutoff voltage of 4.3 V) of Li||NCM811 full cells with the TTE-LHCE electrolyte system; Figure S10. (a–j) Nyquist plots of in situ electrochemical impedance spectra corresponding to different testing stages of the DRT fitting curves during conventional cycling (cutoff voltage of 4.3 V) of Li||NCM811 full cells with the baseline electrolyte (BE) system; Figure S11. Distribution of relaxation time (DRT) curves derived from in situ electrochemical impedance spectroscopy (EIS) measurements of Li||NCM811 full cells with three different electrolytes: (a) TeCA-LHCE electrolyte; (b) TTE-LHCE electrolyte; (c) BE. Each panel displays DRT profiles at different cycling stages (Stage 1 to Stage 10) as a function of relaxation time; Figure S12. Linear sweep voltammetry (LSV) curves of TeCA-LHCE and TTE-LHCE electrolytes; Figure S13. Galvanostatic charge–discharge profiles of Li||NCM811 full cells with three different electrolytes at various cycle numbers: (a) TeCA-LHCE electrolyte; (b) TTE-LHCE electrolyte; (c) BE. All cells were cycled within a voltage window of 2.8-4.3 V vs. Li/Li^+^; Figure S14. (a,b) Charge–discharge curves of Li||NCM811 full cells using TeCA-LHCE and TTE-LHCE electrolytes at various current densities ranging from 0.3 C to 1.5 C.
