# Supplementary material for: Mitigating Interfacial Degradation by Tuning the Diluent–Anion Affinity for Long-Cycling Lithium Metal Batteries
Source: Materials (Basel). 2026 Jun 17;19(12):2605. doi: 10.3390/ma19122605 (PMC13302854; doi:10.3390/ma19122605)
Supplement: Supplementary file 1 [file materials-19-02605-s001.zip › materials-4353147-supplementary.pdf]

Supplementary Materials

Mitigating Interfacial Degradation by Tuning the Diluent–Anion Affinity for Long-Cycling Lithium Metal Batteries

Hongcheng Wu [1,2,†], Jiangnan Ran [1,†], Youxian Dou [2], Dalin Yang [2], Guangye Wu [2,*] and Qiang Zheng [1,*]

1 School of Materials Science and Engineering, Shanghai University, Shanghai 200444, China; hongchengwu@shu.edu.cn (H.W.); 2472996517@shu.edu.cn (J.R.)
2 I-Lab, Suzhou Institute of Nano-Tech and Nano-Bionics, Chinese Academy of Sciences, Suzhou 215123, China; yxdou2025@sinano.ac.cn (Y.D.); dlyang2025@sinano.ac.cn (D.Y.)
* Correspondence: gywu2024@sinano.ac.cn (G.W.); qzheng@shu.edu.cn (Q.Z.)
† These authors contributed equally to this work.

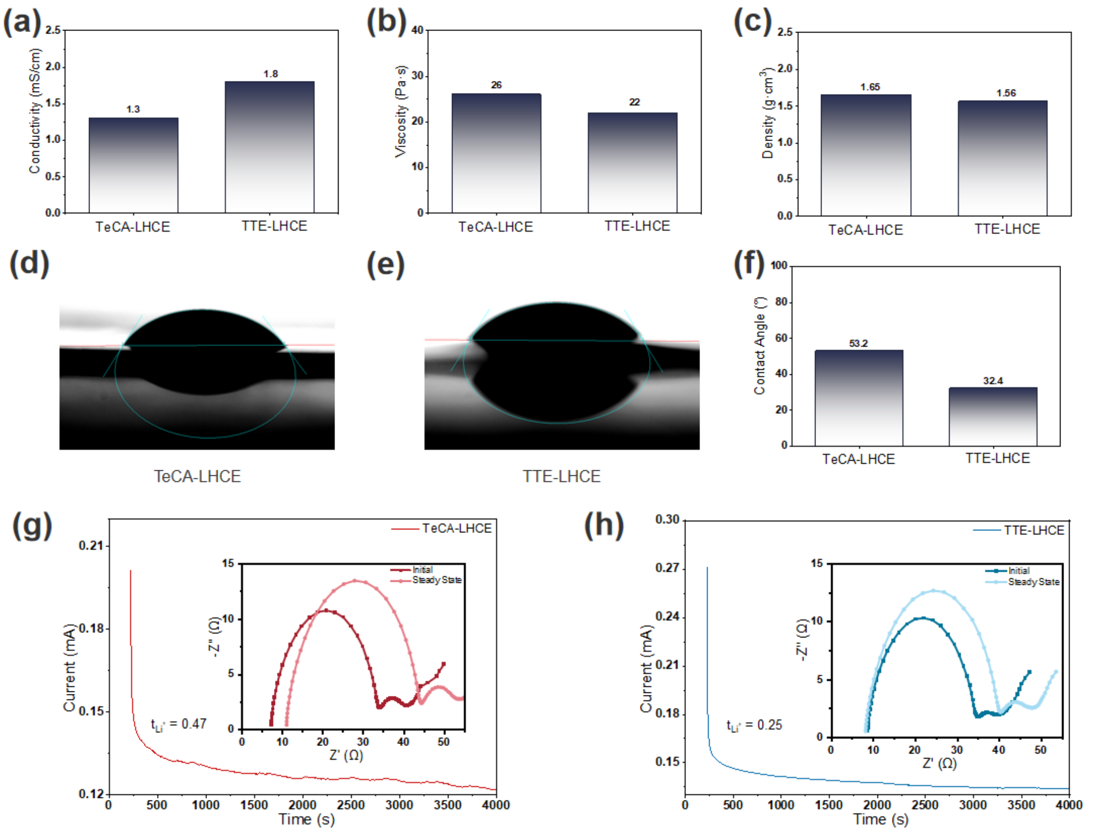

Figure S1. Physicochemical properties and Li⁺ transference numbers of TeCA-LHCE and TTE-LHCE electrolytes. (**a**) Ionic conductivity at 25 °C. (**b**) Viscosity at 25 °C. (**c**) Density at 25 °C. Representative contact angle images of (**d**) TeCA-LHCE and (**e**) TTE-LHCE on the electrode surface. (**f**) Quantified contact angle values. Chronoamperometry curves (main panels) and

corresponding Nyquist plots (insets, before and after DC polarization) of Li symmetric cells with (**g**) TeCA-LHCE and (**h**) TTE-LHCE. The Li^+ transference numbers (t_{Li+}) calculated via the Bruce-Vincent method are labeled in each plot.

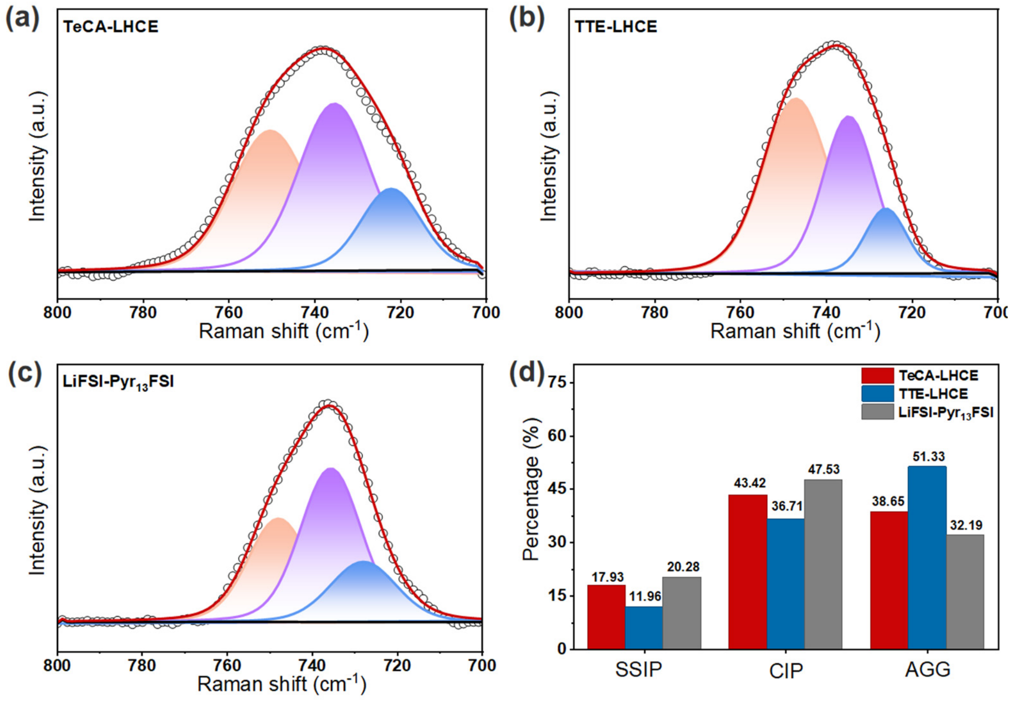

Figure S2. Raman spectroscopy analysis of solvation structures in different electrolytes. Deconvoluted Raman spectra for (**a**) TeCA-LHCE, (**b**) TTE-LHCE, and (**c**) LiFSI-Pyr_{13}FSI electrolytes. The spectra are fitted into three components corresponding to solvent-separated ion pairs (SSIP), contact ion pairs (CIP), and aggregates (AGG). (**d**) Relative percentages of SSIP, CIP, and AGG derived from peak deconvolution for the three electrolyte systems.

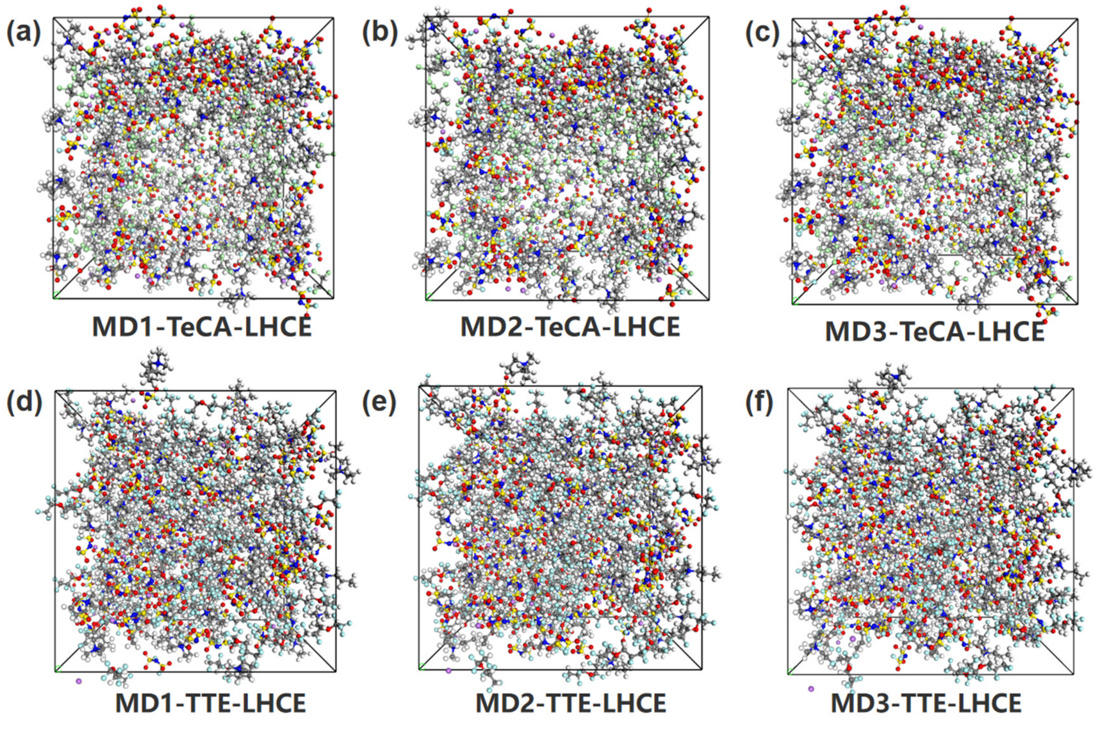

Figure S3. Molecular dynamics (MD) simulation snapshots of equilibrated electrolyte systems. (**a-c**) Three independent parallel simulation runs with the TeCA-LHCE electrolyte. (**d-f**) Three independent parallel simulation runs with the TTE-LHCE electrolyte.

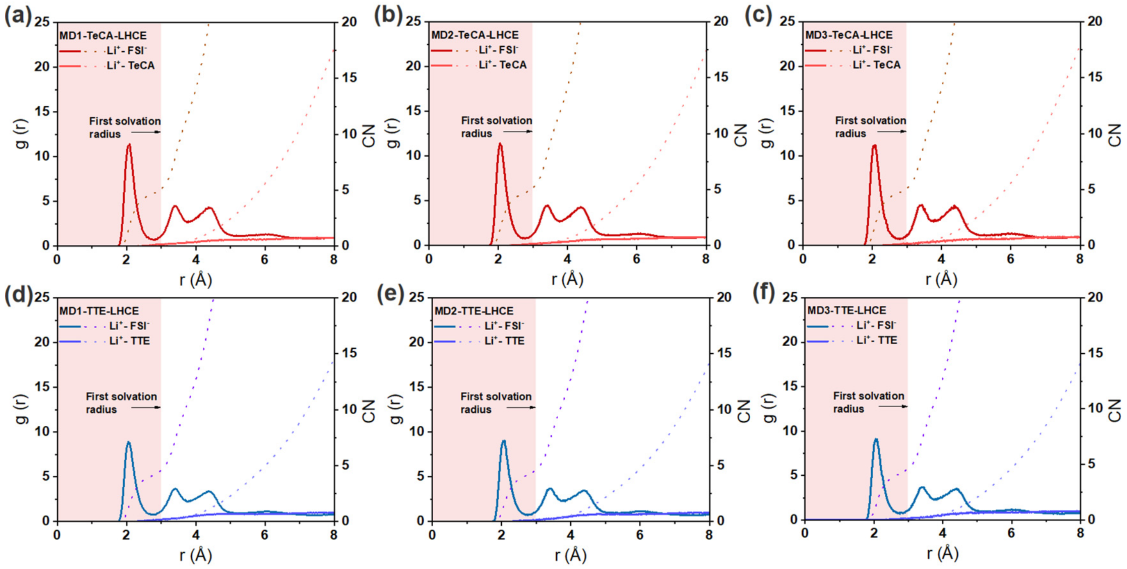

Figure S4. Radial distribution functions (RDFs) and corresponding coordination numbers (CNs) from three independent molecular dynamics (MD) simulations. (**a-c**) RDF and CN for Li^+-FSI^- and Li^+-TeCA in the TeCA-LHCE electrolyte. (**d-f**) RDF and CN for Li^+-FSI^- and Li^+-TTE in the TTE-LHCE electrolyte. The pink shaded region denotes the first solvation radius of Li^+.

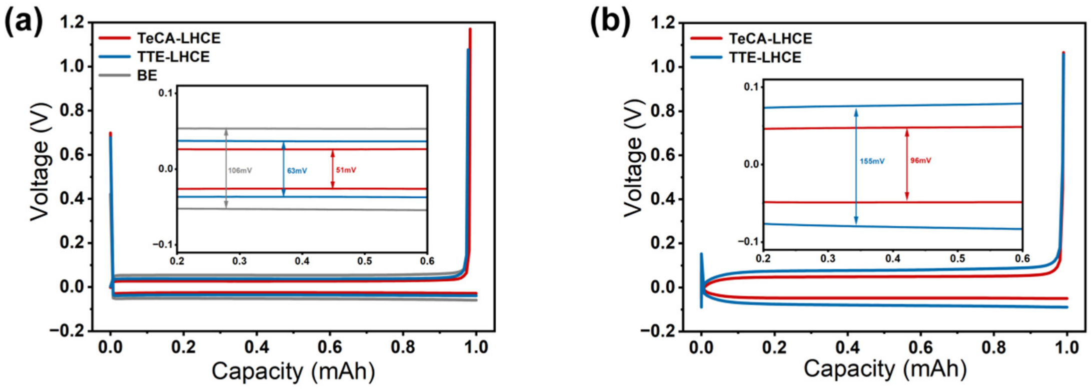

Figure S5. (**a, b**) Galvanostatic lithium plating/stripping voltage profiles of Li||Cu cells in the TeCA-LHCE, TTE-LHCE, and baseline electrolyte (BE) systems at the 100th and 200th cycles, respectively.

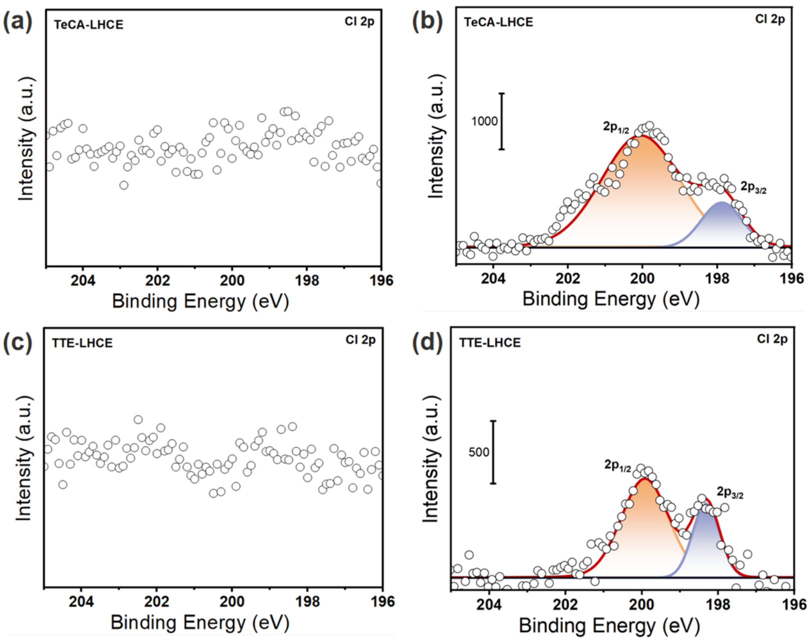

Figure S6. XPS Cl 2p characterization of cycled anodes and cathodes in TeCA-LHCE and TTE-LHCE electrolytes. (**a**) Unfitted raw Cl 2p spectrum of a cycled anode from the TeCA-LHCE system. (**b**) Deconvoluted Cl 2p spectrum of a cycled cathode from the TeCA-LHCE electrolyte, showing resolved spin–orbit split $2p_{1/2}$ and $2p_{3/2}$ peaks. (**c**) Unfitted raw Cl 2p spectrum of a cycled anode from the TTE-LHCE system. (**d**) Deconvoluted Cl 2p spectrum of a cycled cathode from the TTE-LHCE electrolyte, with resolved $2p_{1/2}$ and $2p_{3/2}$ peaks.

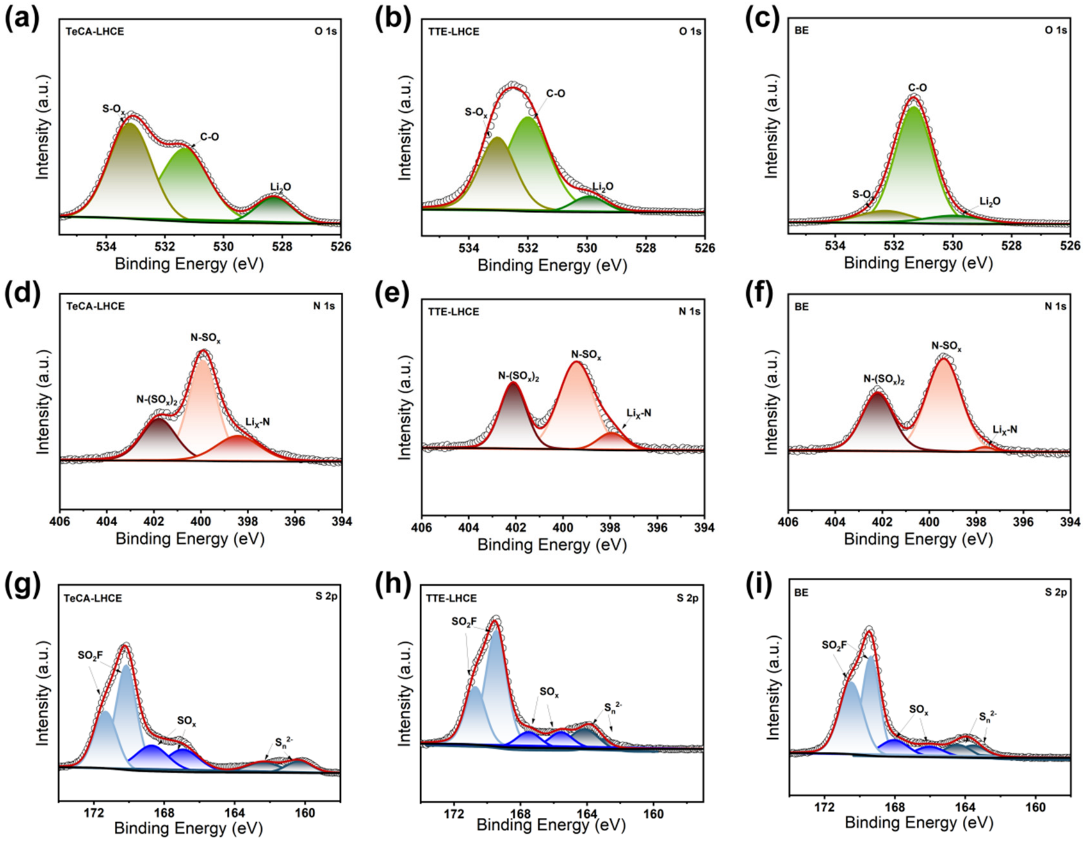

Figure S7. (**a-c**) Fitted O 1s XPS spectra of the NCM811 cathode surfaces in the BE, TTE-LHCE, and TeCA-LHCE systems, respectively. (**d-f**) Corresponding N 1s XPS spectra of the three electrolyte systems. (**g-i**) Fitted S 2p XPS spectra of the three electrolyte systems.

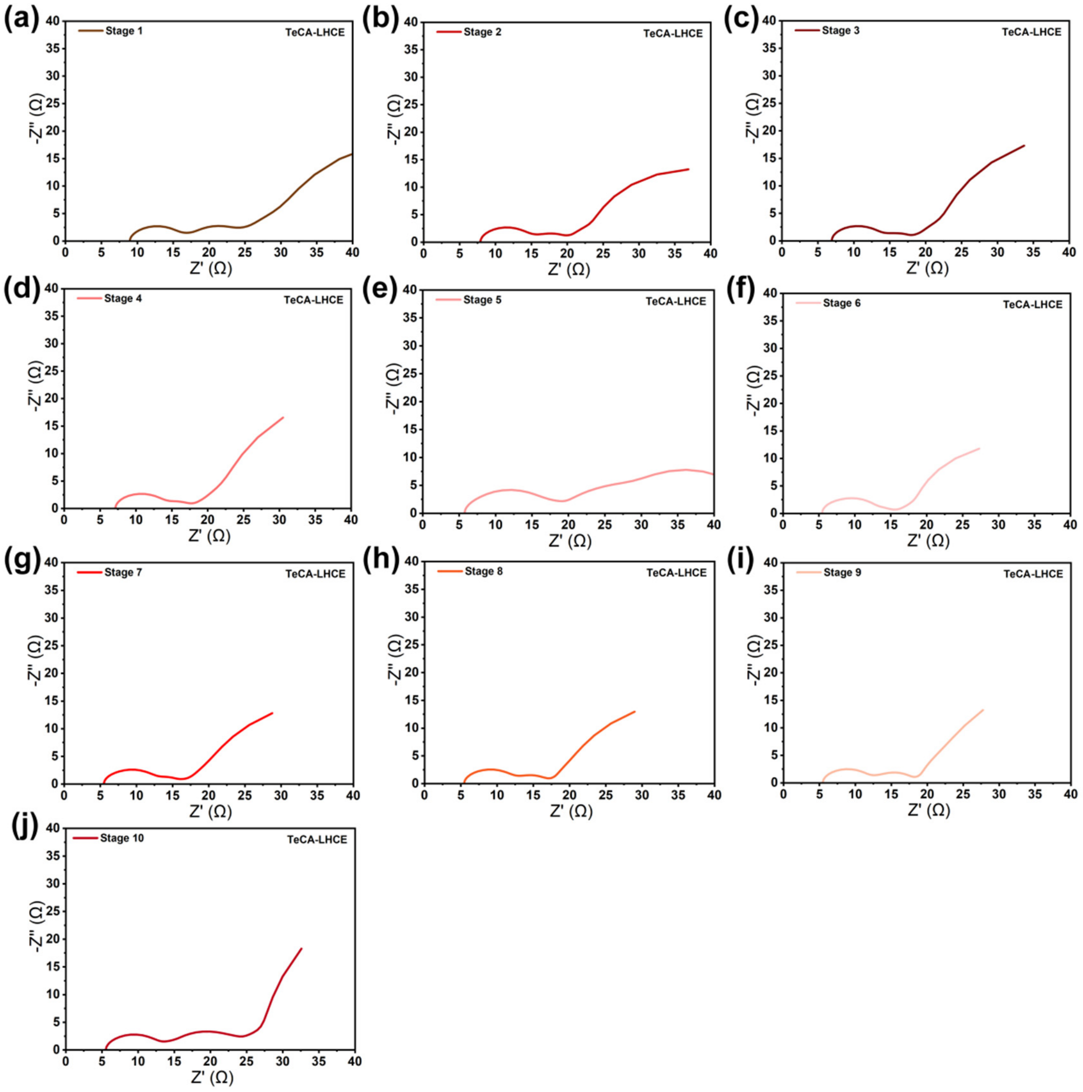

Figure S8. (**a-j**) Nyquist plots of in situ electrochemical impedance spectra corresponding to different testing stages of the DRT fitting curves during conventional cycling (cutoff voltage of 4.3 V) of Li||NCM811 full cells with the TeCA-LHCE electrolyte system. The spectra consist of a semicircle in the high-frequency region, corresponding to interfacial contact and charge-transfer resistance, and an inclined line in the low-frequency region, corresponding to the solid-state ion diffusion process.

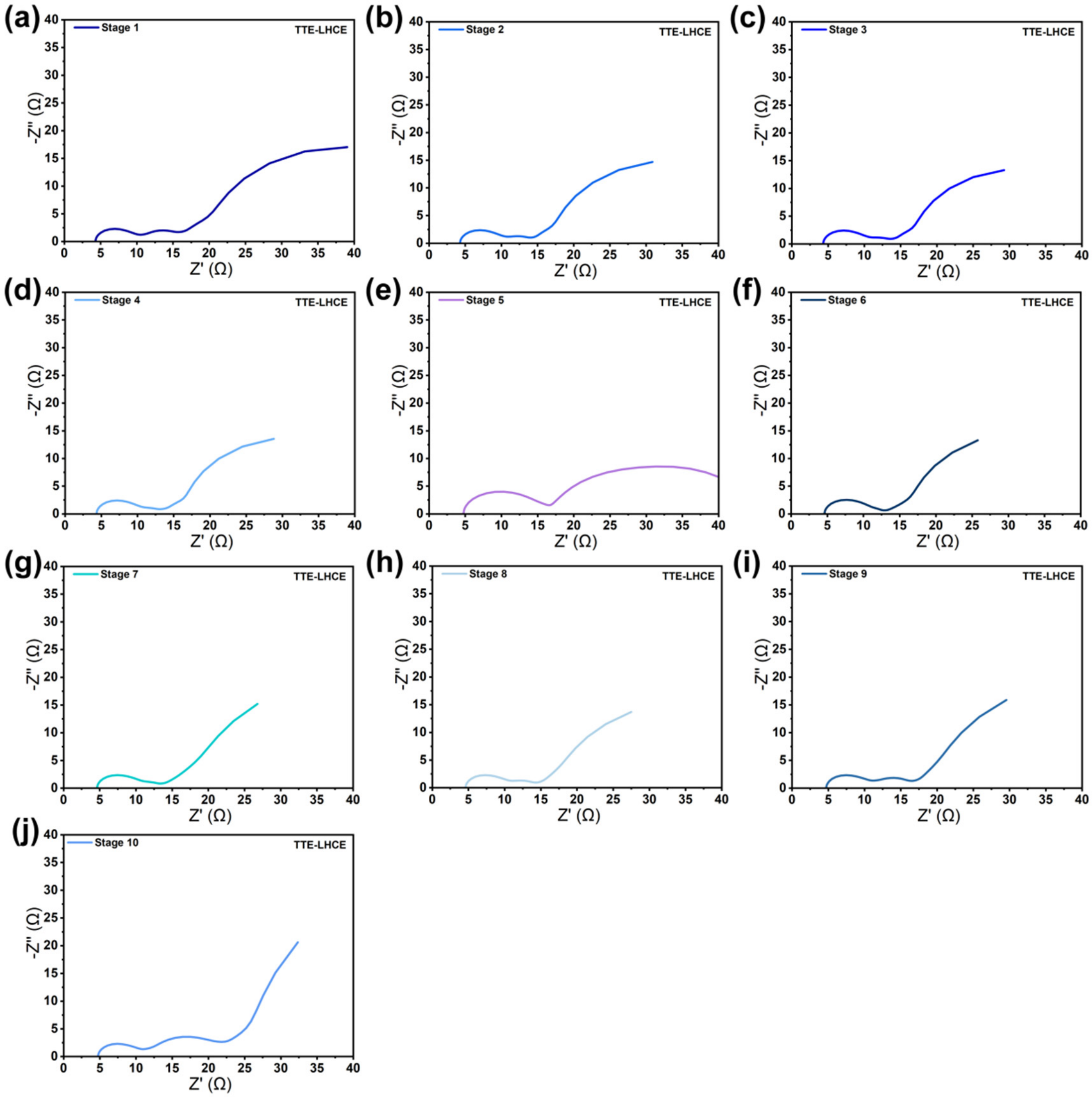

Figure S9. (**a-j**) Nyquist plots of in situ electrochemical impedance spectra corresponding to different testing stages of the DRT fitting curves during conventional cycling (cutoff voltage of 4.3 V) of Li||NCM811 full cells with the TTE-LHCE electrolyte system.

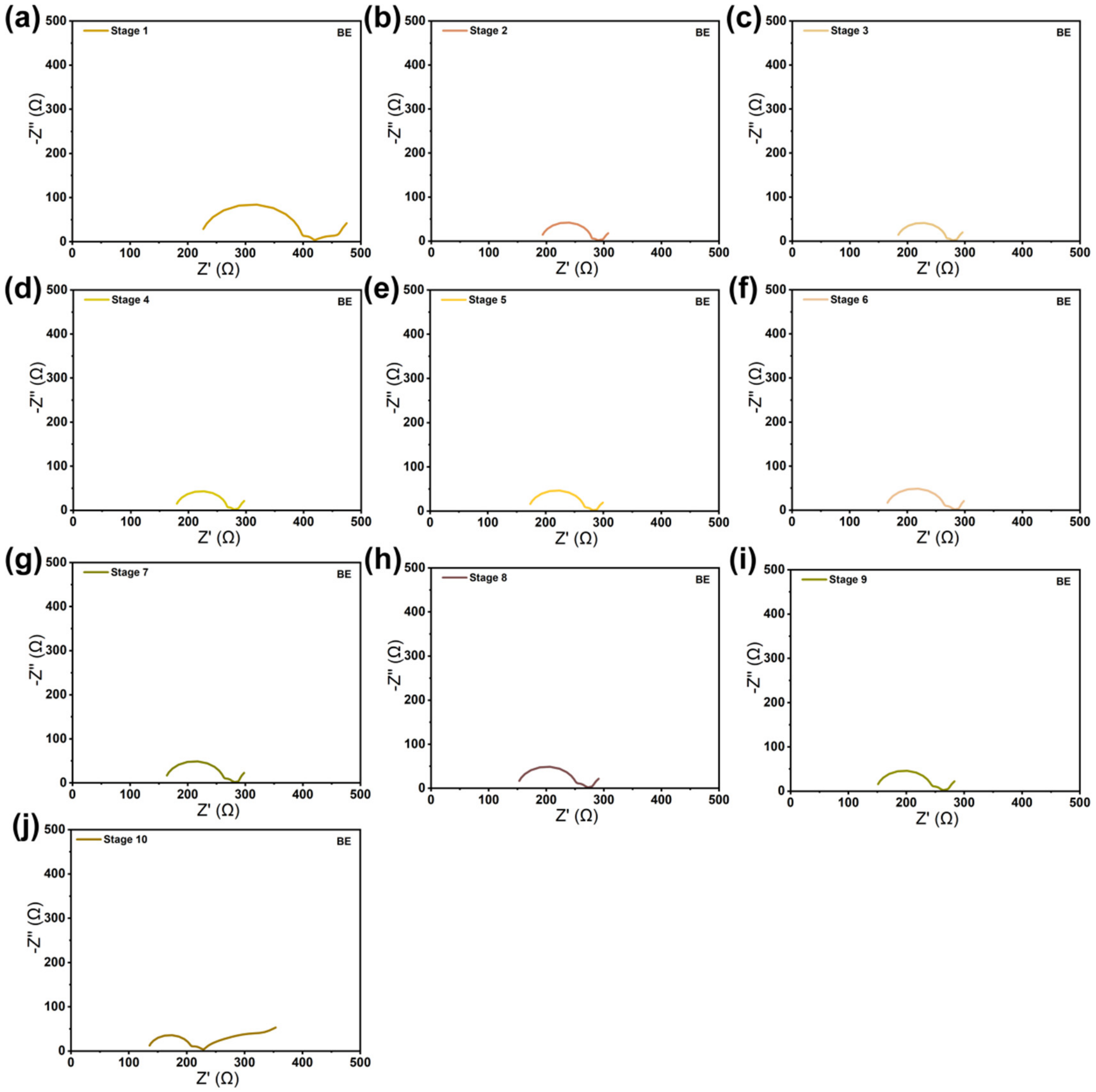

Figure S10. (**a-j**) Nyquist plots of in situ electrochemical impedance spectra corresponding to different testing stages of the DRT fitting curves during conventional cycling (cutoff voltage of 4.3 V) of Li||NCM811 full cells with the baseline electrolyte (BE) system.

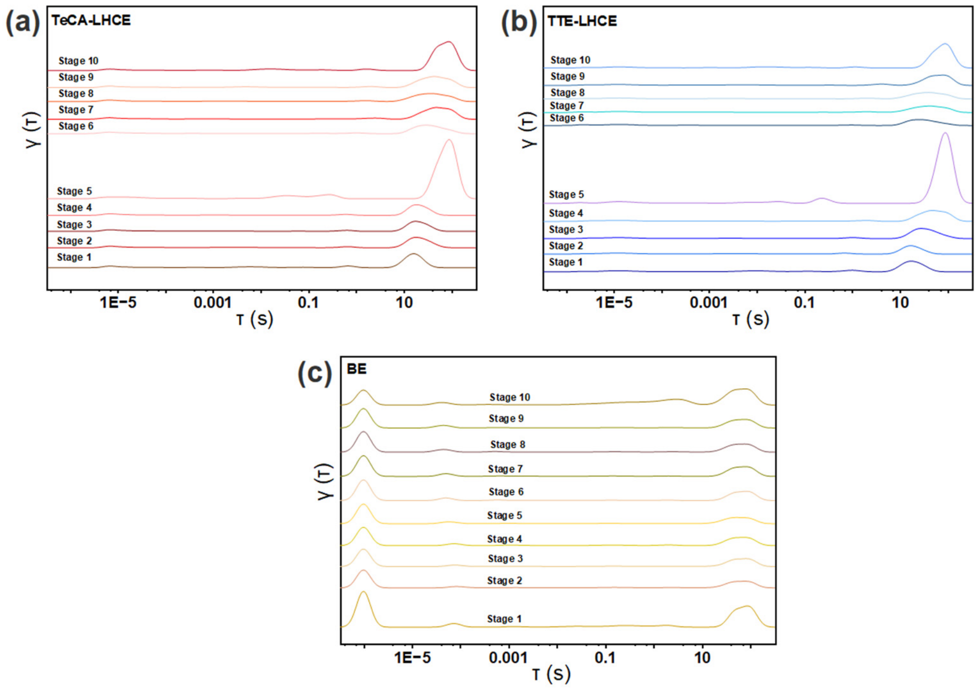

Figure S11. Distribution of relaxation time (DRT) curves derived from in situ electrochemical impedance spectroscopy (EIS) measurements of Li||NCM811 full cells with three different electrolytes: (**a**) TeCA-LHCE electrolyte; (**b**) TTE-LHCE electrolyte; (**c**) BE. Each panel displays DRT profiles at different cycling stages (Stage 1 to Stage 10) as a function of relaxation time.

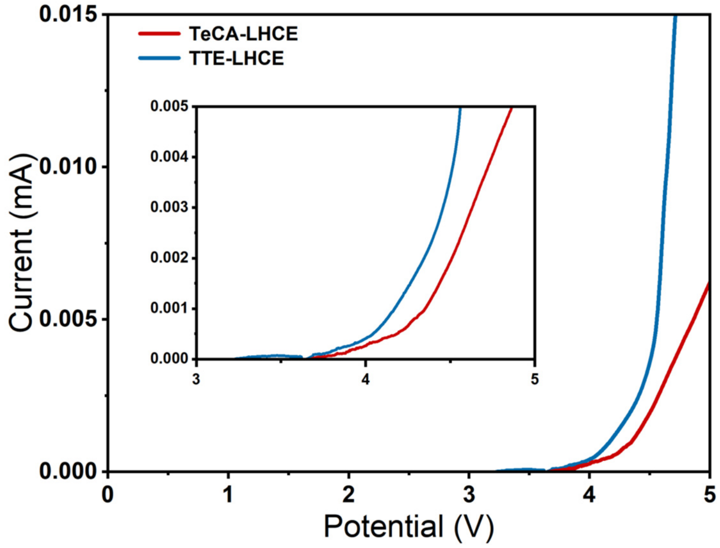

Figure S12. Linear sweep voltammetry (LSV) curves of TeCA-LHCE and TTE-LHCE electrolytes.

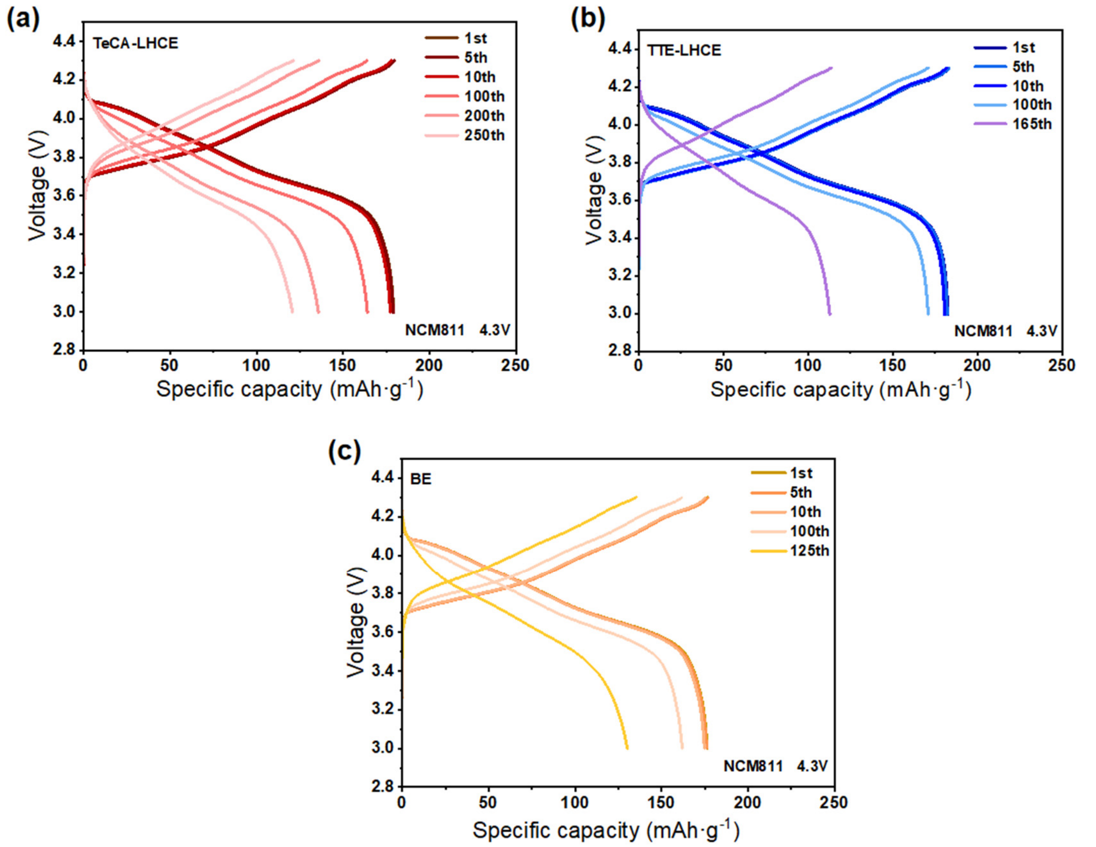

Figure S13. Galvanostatic charge–discharge profiles of Li||NCM811 full cells with three different electrolytes at various cycle numbers: (**a**) TeCA-LHCE electrolyte; (**b**) TTE-LHCE electrolyte; (**c**) BE. All cells were cycled within a voltage window of 2.8-4.3 V vs. Li/Li^+.

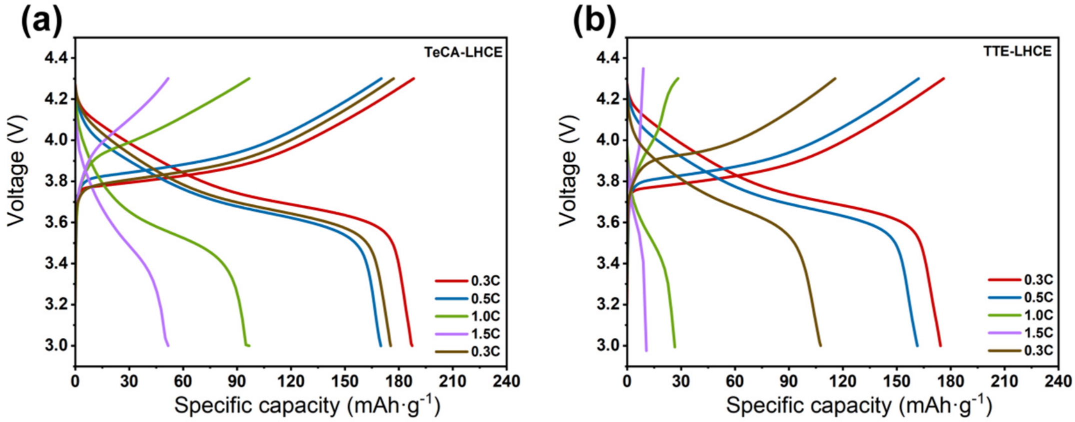

Figure S14. (**a, b**) Charge–discharge curves of Li||NCM811 full cells using TeCA-LHCE and TTE-LHCE electrolytes at various current densities ranging from 0.3 C to 1.5 C.
